# Analysis of the Local Agenda 21 in Madrid Compared with Other Global Actions in Sustainable Development

**DOI:** 10.3390/ijerph16193685

**Published:** 2019-09-30

**Authors:** Jose Manuel Diaz-Sarachaga

**Affiliations:** GTDS Research Group, Universidad de Oviedo, C/Catedratico Valentin Andres Alvarez s/n, 33006 Oviedo, Spain; jmdsarachaga@hurbannia.com

**Keywords:** Local Agenda 21, Spanish urban agenda, sustainable urban development, sustainable development goals, public participation

## Abstract

Over the last two decades, numerous towns have been involved in the Local Agenda 21 program in Spain, which is founded on social participation. In the wake of this initiative, the recent promotion of the new Spanish Urban Agenda by the national government seeks to implement the 2030 Agenda in municipalities nationwide. This research aims to examine the Local Agenda 21 process by using Madrid as a case study to determine the lessons learned to enable the effective application of the new Spanish Urban Agenda. A total of 3712 activities included in the action plans of the 21 districts of Madrid were analyzed to identify linkages with the Sustainable Development Goals and the targets of Sustainable Development Goal # 11 (“Sustainable cities and communities”). Methodologies used were solely oriented to develop an ad hoc Local Agenda 21 plan for each district, hindering the comparison of schemes and findings. Social, institutional, economic, and environmental dimensions of sustainable development were not equally considered by the plans, being the first two aspects the most predominant. Social engagement hardly reached 0.44% of the registered population. The contribution of all action plans to the sustainable development of Madrid was not assessed due to the absence of indicators in the program.

## 1. Introduction

In June 2018, the Spanish government approved the action plan for the implementation of the 2030 Agenda [1]. The new Spanish Urban Agenda (SUA) [2] thus arose as the most relevant policy to boost the deployment of the Sustainable Development Goals (SDGs) in the country to end poverty, protect the environment, and ensure widespread peace and security for all individuals [3]. A four-fold approach covering social, economic, environmental, and institutional dimensions is used by the SUA to face challenges derived from the inexorable growth of urban population as a consequence of the abandonment of rural areas by young people, namely the increasing need of habitable land and decent housing, social inequality, the loss of cultural identity or the effects of climate change, among others. In this vein, the development of agendas at the local scale is crucial to incorporate the daily concerns of citizens by involving municipal governments as well as other public and private stakeholders in order to launch actions with the aim of enhancing the quality of life for inhabitants in urban areas [4]. 

As a pioneering initiative held in Rio in 1992, the UN Conference on Environment and Development (UNCED), otherwise known as the Earth Summit, represented a commitment for the international community to harmonize economic growth and environmental preservation. Agenda 21 [5] was adopted to achieve sustainable development worldwide. The essential role of local administrations concerning the realization of sustainability objectives was recognized in chapter 28 of Agenda 21, entitled “Local Authorities’ Activities in Support of Agenda 21”, where their participation was triggered by means of Local Agenda 21 (LA21), a comprehensive framework on the basis of public involvement, open decision-making structures, multi-sectorial partnerships, and cross-border networking [6] to translate the principles and mandates of Agenda 21 into concrete strategies for each local community.

The signature of the Aalborg Charter in 1994 [7], under the auspices of the European Commission and the International Council for Local Environment Initiatives (ICLEI), served to launch the European Sustainable Cities and Towns Campaign inspired by the LA21 plan to strengthen urban sustainability. The Charter was formulated by gathering insights from citizens, local authorities, academia, and organizations at national and international levels. ICLEI presented the Local Agenda 21 Planning Guide in 1996 to assist municipalities in the management of human settlements toward sustainability under the consideration of the Agenda 21 action plan [8]. For this purpose, the design of sustainable development strategies should be addressed to cope with systemic problems in the long term by engaging all relevant stakeholders and using the equal weighting of economic, social and environmental aspects [9]. The Planning Guide is comprised of five elements: partnerships, community-based issue analysis, action planning, implementation and monitoring, and evaluation and feedback. In June 2004, the 4th European Conference on Sustainable Cities and Towns released a framework covering 50 qualitative goals organized into 10 comprehensive topics known as the Aalborg Commitments (AC) [10] to foster the local efforts on sustainability and revitalize LA21. Sustainable development was thus deemed in a holistic manner to solve the problems of mankind [11] and impede the destruction of the planet [12] by embracing diverse European policies such as the Lisbon Strategy, the Sixth Environment Action Program, and the forthcoming Strategy on the Urban Environment, along with the Millennium Development Goals and the Johannesburg Plan of Implementation.

Findings of a survey conducted by ICLEI in 2002 to identify the level of LA21 activity worldwide revealed the participation of 6416 local governments in 113 countries. Europe reflected the highest number of municipalities involved in the process (5292) representing 36 nations, followed by Asia with 674 local agendas implemented in 17 countries. Africa, Latin America, and North America undertook more than 100 national campaigns each for 28, 17, and 2 countries, respectively. In the Middle East, 79 processes underway were reported in 13 countries [13]. Germany (2042), the United Kingdom (425), Italy (429), and Spain (359) led the ranking of countries, but their time of reaction differed substantially [14]. Whilst the United Kingdom boosted LA21 processes from 1992, Germany promoted LA21 efforts after 1997 [15,16]. Italy and Spain began the deployment of LA21 in the early 2000s [17].

Distinct aspects of LA21 have been covered in the literature. The amount of resources assigned to local governments was repeatedly highlighted as an outstanding factor to promote the wide diffusion of LA21 [18]. The dynamic nature of sustainability over the long term was reflected in LA21 [19,20]. Some deficiencies at the municipal level arising from inadequate organizations, short term vision, scant training on sustainability for politicians, and the lack of interdisciplinary approaches impacted significantly on LA21 [21]. In most municipalities, LA21 was not regarded as an instrument of strategic planning that incorporated the transversal concept of sustainability [22].

The significant role of Spain as one of the major players worldwide in the process of LA21 and the forthcoming application of the SUA, which follows the same methodology, were the main reasons for undertaking this study. The definition of areas where improvements are needed to enable the successful launch of the new initiative was the aim of the research. Sustainable Development Goals valid until December 2030 were established as the benchmark to analyze the Local Agenda 21 enforced in Madrid. 

The structure of the manuscript encompasses three additional sections. First, the research method and LA21 process in Madrid are described in Section 2. Section 3 presents the results obtained from the study and their discussion are the object of Section 4. Finally, the main conclusions of the article are summarized in Section 4. 

## 2. Methodology and Materials 

In this section, a brief overview of the stages followed in the research is described. The process of LA21 in Madrid is presented in detail as follows.

### 2.1. Methodology 

**Figure 1:**
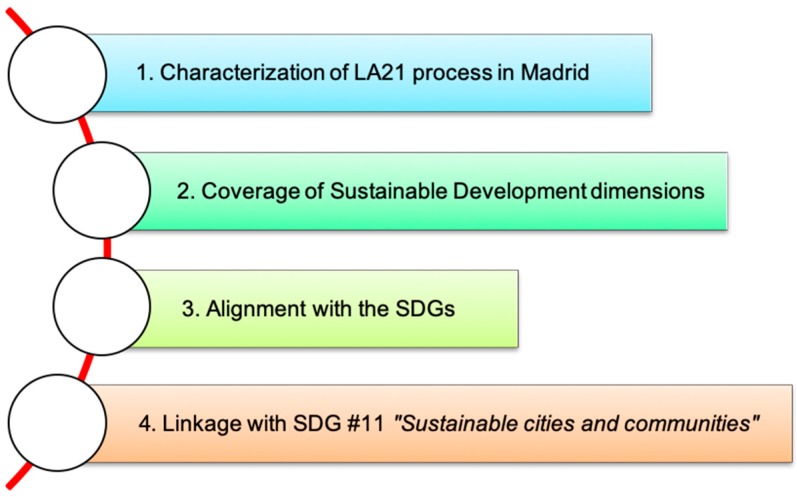
Methodology of the research (Source: author). SDGs: Sustainable Development Goals. LA21: Local Agenda 21. SDG # 11: Sustainable cities and communities.

Figure 1 shows the methodology defined for the study. After depicting the Local Agenda 21 (LA21) implemented in Madrid, all actions entailed in the action plan were analyzed for correlation with all of the sustainable development dimensions, namely social, economic, environmental, and institutional. Furthermore, these actions were categorized as per their correspondence with the 17 Sustainable Development Goals (SDGs) and the 10 targets deemed in SDG # 11 (“Sustainable cities and communities”). 

### 2.2. Local Agenda 21 in Madrid

Madrid City Council subscribed the Aalborg Charter in 1996 and adopted the 10 Aalborg Commitments toward sustainable development in 2007 [23]. The implementation process of LA21 in the 21 boroughs of Madrid commenced in 2003. Commissions of Agenda 21 were established in the territorial councils of each district to ensure the participation of the citizenry. The Code of Good Environmental Practice championed by the Spanish Federation of Municipalities and Provinces (FEMP) [24] served to conduct LA21 in three phases. An initial prognosis was performed during the first stage to determine the needs and strengths of the boroughs as perceived by their inhabitants. In the second phase from 2006 to 2008, an action plan was developed for each district of the city. The formulation of specific action plans for each single borough was intended to gather the concerns, needs and suggestions from local residents in a more precise way, although the actions selected were not cross-connected with those of other districts. Drafts were released for public comment to be then approved by each LA21 standing commission. A total of 3712 actions were contemplated by all LA21 action plans. The last step began in April 2008, which was to monitor and evaluate the progress of action plans by engaging borough councils. The final progress report was launched in March 2013.

No efforts in sustainable urban development had been previously undertaken in Madrid before the application of LA21, which can be framed as a one-off initiative for a particular time period due to the lack of a national framework with common guidelines for all Spanish municipalities. Although several global policies and strategies were adopted during the life of LA21 in Madrid, all action plans remained as they were initially conceived. Moreover, the performance of LA21 action plans coincided in time with the acute financial crisis suffered by Spain, which resulted in severe budget cuts. However, the unavailability of a timeline to complete the actions envisaged prevents the evaluation of the impacts derived from the economic downturn in the application of the LA21 process in Madrid.

LA21 was then replaced by the Integrated Sustainable Urban Development Strategies as part of the legislative proposals for cohesion policy adopted by the European Commission in 2011. Under the European Regional Development Fund (ERDF) regulation, the 2007–2013 period provided the option of implementing urban development with an integrated approach (Article 8), whilst strategies covering integrated actions could be operationalized from 2014 to 2020 (Article 7). At the very least, 5% of the ERDF resources were allocated to be invested by cities in actions for sustainable urban development over this period [25]. 

Diverse documents for every one of the 21 districts of Madrid city are available from the Municipality of Madrid website such as the sustainability assessment, public consultation report, action plan, and final progress report [26]. Action plans were devised by each LA21 standing commission, which is composed of representatives of political parties, neighborhood associations, and other kind of civic organizations as well as individuals. Six main themes shaped the common structure of the action plans for all boroughs: urban layout, economic growth, natural resources and urban environment, basic infrastructure and housing, labor market and community service, and public participation. Training and education on sustainable urban development for residents, local officials, and politicians remained uncovered. 

**Figure 2:**
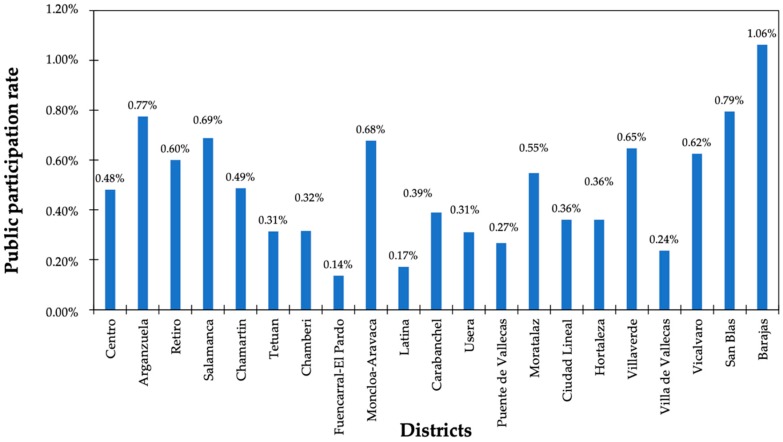
Public participation rate per district [26].

Beyond the representation of citizens in LA21 standing commissions, the public engagement was limited to express an opinion on diverse subjects such as employment, environment, movement and transport, and urbanism during the diagnosis stage and the public consultation exercise on the drafts of action plans. Figure 2 exhibits the percentages of public participation in this manner. Although the registered population in Madrid amounted to 3,177,587 inhabitants, only 13,924 questionnaires were collected via the ballot box or electronic means. In short, only 0.44% of the census was implicated in the development of action plans. Barajas reflected the highest rate of civil engagement (1.06%); in comparison, the Fuencarral-El Pardo and Latina districts showed the lowest contribution by 0.14 and 0.17%, respectively. These figures are in contrast with some studies that have indicated that the proportion of residents involved in LA21 processes is close to 1% [27]. 

**Figure 3:**
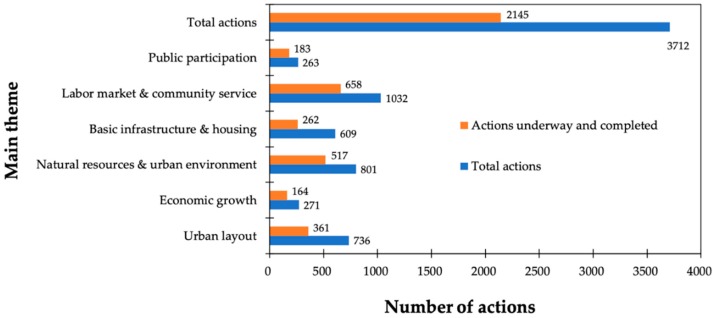
Distribution of the actions by the LA21 thematic areas [26].

The analysis of the 3712 actions deemed in the 21 action plans revealed that most of them (2910) were associated to specific departments of the city hall, whilst 710 were governed by the borough councils and 92 were dependent on the neighbors. Hence, the role of citizens in the management of selected actions was also not relevant. Figure 3 illustrates the distribution of all actions according to one of the six themes that form the LA21 in Madrid and distinguishes between the actions in progress and completed (58%), and the total amount. Labor market and community service (1032) were the subjects with the highest number of actions in comparison with the lowest quantity of public participation (263). With regard to ongoing and completed actions, the trend was similar, with predominating actions connected with the labor market and community service (658). In contrast, economic growth only reflected 164 actions. 

**Figure 4:**
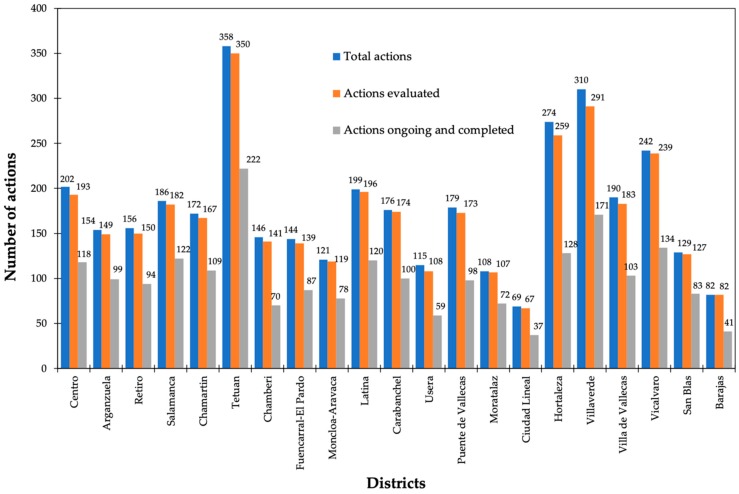
Actions evaluated, underway and completed in boroughs in Madrid [26].

The progress report of the action plans omits any metrics to assess the contribution of efforts in the sustainable urban development of each district. Only the status of execution was examined by determining three levels of achievement only applicable to the actions evaluated: ongoing or completed, unexecuted, and without information. The shortage of information regarding the degree of feasibility or definition about its scope/goal were the criteria to label an action as “pending evaluation”. The lack of both quantitative and qualitative goals to be achieved at the completion of the action plans and an initial baseline to measure the advances are serious shortcomings to assess the effectiveness of the plans. The allocation of LA21 actions by the boroughs ascertained that the action plan of Tetuan gathered the largest number of initiatives (358) as well as the highest number of actions evaluated (350) and ongoing/completed (222). Conversely, Ciudad Lineal displayed the worst records. From the total actions (69), 67 were assessed, but only 37 had been finished or were in progress (Figure 4).

## 3. Results and Discussion

The relationship between all efforts considered in the 21 action plans of Madrid and the sustainability dimensions, the 17 SDGs, and the 10 targets covered in the SDG # 11 is examined in this section. The findings are shown as follows. 

### 3.1. Liaison with the Sustainability Dimensions 

Although Elkington [28] originally associated sustainable development with social, economic, and environmental facets, other authors have later incorporated the institutional aspect [29]. The balance between all those dimensions is necessary to achieve sustainable development [30]. Under this premise, the 3712 initiatives of the 21 action plans implemented in the city of Madrid were grouped according to their correspondence with the sustainability dimensions (Table 1). 

**Figure 5:**
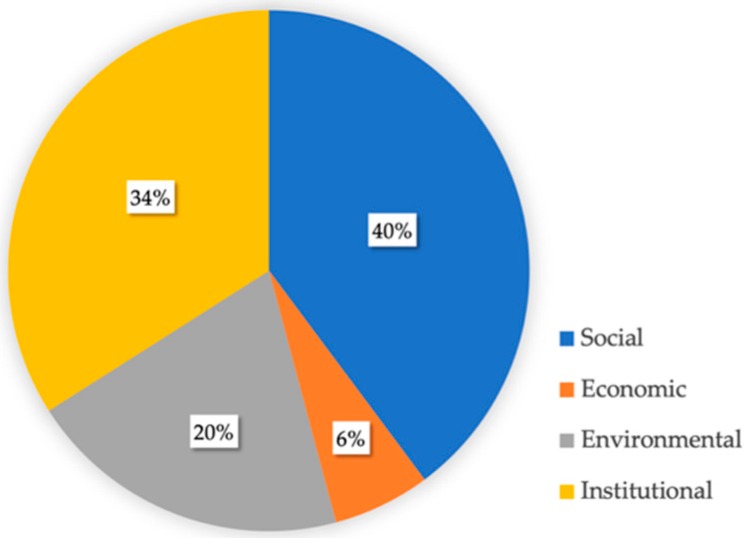
Breakdown of actions by sustainability dimension (Source: author).

The final objective of sustainability is not so much the priority treatment of any of its social, economic, environmental, or institutional components, but to strengthen the whole [31]. Nevertheless, LA21 often shows a strong bias toward a single dimension, while the remainder are more or less neglected [32]. In the case of Madrid, social (40%) was the aspect that was most addressed by the boroughs, followed by the institutional (34%) and environmental dimensions (20%). Efforts oriented to stimulate urban economic growth in the long term was only recorded as 6% of the total (Figure 5). Despite the LA21 process in Madrid was led by the environmental department of the city council, no preference for that dimension was viewed in contrast to the other processes in Europe [33].

The number of initiatives, size, population, and public participation were the four approaches that served to determine the patterns and correlations between the districts and sustainability dimensions. As displayed in Table 1, the boroughs with a larger number of actions in their plans reflected, in general, the highest ratios of linkage in the social, economic, and environmental aspects and vice versa. That tendency reverses for the institutional aspect. The size of the districts was directly proportional to the percentage of actions connected with the social dimension, and indirectly proportional to those tied to the economic aspect. No pattern was revealed for the environmental and institutional aspects. Neither population nor civil engagement were determinant to define any trend that involved the four sustainability pillars. 

**Figure 6:**
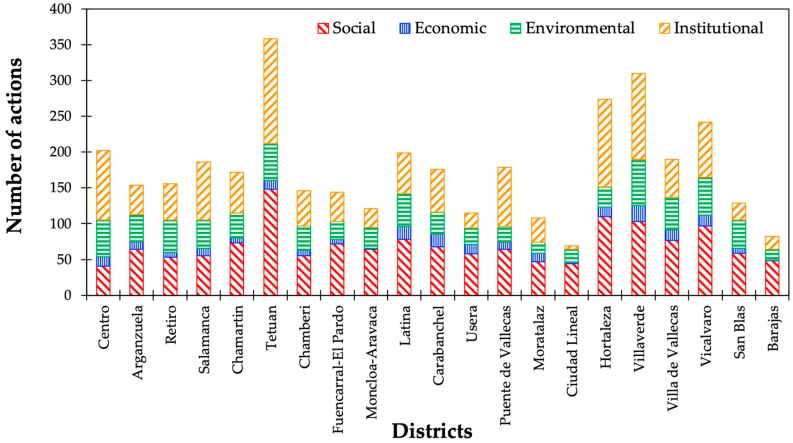
Categorization of actions by borough and sustainability dimension (Source: author).

As illustrated in Figure 6, a district-based analysis showed that social and institutional dimensions were mostly covered by Tetuan, whereas Villaverde accounted for the largest number of actions in the economic and environmental fields. On the other hand, Barajas showed the fewest efforts in the economic and environmental aspects. Centro and Ciudad Lineal were hardly concerned with social and institutional issues. 

### 3.2. Alignment with the Sustainable Development Goals 

Efforts by the international community for a sustainable world in 2030 are focused on achieving the SDGs (Table 2) adopted in the United Nations Sustainable Development Summit held in 2015 [34]. This global initiative aims at encouraging low, medium, and high-income countries to take steps toward economic prosperity while protecting the planet. The SDGs also recognize that the end of poverty demands a combined strategy that encompasses economic growth and the coverage of basic needs in education, health, social welfare, and employment as well as the importance of the fight against climate change and the preservation of the environment [35]. As the 17 SDGs replaced the Millennium Development Goals that were considered in the definition of the 10 Aalborg Commitments, the connection between those and the SDGs also needed to be disclosed (Table 2). 

**Figure 7:**
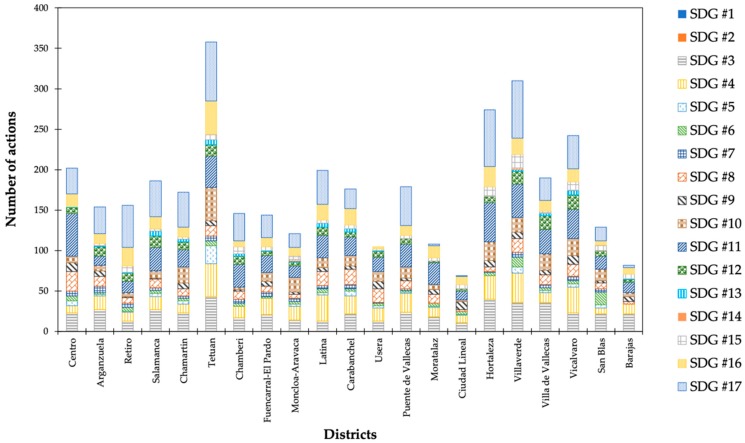
Assignment of actions by borough and SDG (Source: author).

SDGs # 3 (“Good health and well-being”), # 4 (“Quality education”), # 10 (“Reduced inequalities”), # 13 (“Climate action”), and # 16 (“Peace, justice and strong institutions”) were mainly considered in Tetuan. Centro reflected a larger number of actions linked to the SDG # 1 (“No poverty”), # 8 (“Decent work and economic growth”), # 9 (“Industry, innovation and infrastructure”), and # 11 (“Sustainable cities and communities”). The SDGs # 9 (“Industry, innovation and infrastructure”) and # 12 (“Responsible consumption and production”) gathered the most actions in Vicalvaro, similar to Villaverde with SDGs # 14 (“Life below water”) and # 15 (“Life on land”). Arganzuela, Retiro, Salamanca, Moncloa, and San Blas followed suit with SDGs # 7 (“Affordable and clean energy”), # 17 (“Partnerships for the goals”), # 13 (“Climate action”), # 14 (“Life below water”), and # 6 (“Clean water and sanitation”) (Figure 7).

Attempts made by local authorities to promote sustainable practices in municipalities have not been sufficient [36,37], which is the reason why “Partnerships for the goals” (SDG # 17) disclosed the highest number of initiatives, followed by “Sustainable cities and communities” (SDG # 11) and “Good health and well-being” (SDG # 3), respectively. On the other hand, “No poverty” (SDG # 1) and “Zero hunger” (SDG # 2) hardly garnered any actions (Table 3). This fact is highly remarkable, since both goals represent the main themes on which the Millennium Development Goals (2001–2015) and the SDGs (2016–2030) were based. Furthermore, those figures conceal the effects of the acute financial crisis faced by Spain primarily in the 2007–2013 period such as a cumulative 8.9 per cent contraction of gross domestic product and a growth of the unemployment rate from 7.8 to 27.2%. More than three million people left the middle class as a result, causing a substantial increase in the level of poverty [38].

Education (SDG # 4), employment (SDG # 8), income inequalities (SDG # 10), and strong institutions (SDG # 16) subjects were dealt by a significant number of actions. Gender equality (SDG # 5), basic services (SDG # 6 and # 7), and climate (SDGs # 13–# 15) themes, in contrast, collected fewer initiatives. 

The small number of actions mostly relating to poverty, hunger, and gender equality issues might be due to the ongoing projects linked to the social dimension that are typically conducted by non-governmental organizations and charities over time in the framework of their core activities and beyond initiatives such as the LA21 process. These projects are normally aligned with local policies regarding the matter.

The distribution of the 3712 actions between the 10 Aalborg Commitments adopted by Madrid in 2004 was also uneven. AC # 1 (“Governance”) and # 2 (“Local management towards sustainability”) were both very poorly represented. However, ACs # 3 (“Natural common goods”) and # 4 (“Responsible consumption and lifestyle choices”) had the highest number of initiatives. The remaining commitments reflected an intermediate number of actions. Therefore, in light of the above, it can be stated that the action plans of the 21 districts of Madrid were not designed under the consideration of the full coverage of the Aalborg Commitments [39]. 

### 3.3. Linkage with the Targets of SDG # 11 (“Sustainable Cities and Communities”)

The nature of SDG #11 is markedly urban, as attested by its primary aim of making cities safer and more inclusive, sustainable, and resilient. Their 10 targets, as shown in Table 4, were thereby taken as the benchmark to examine the initiatives of all of the action plans. The relationship between the targets and actions contemplated in each of the plans proposed by the 21 boroughs of Madrid is exhibited in Table 5. Less than 20% of the 3712 actions revealed any connection to the targets of SDG # 11. 

**Figure 8:**
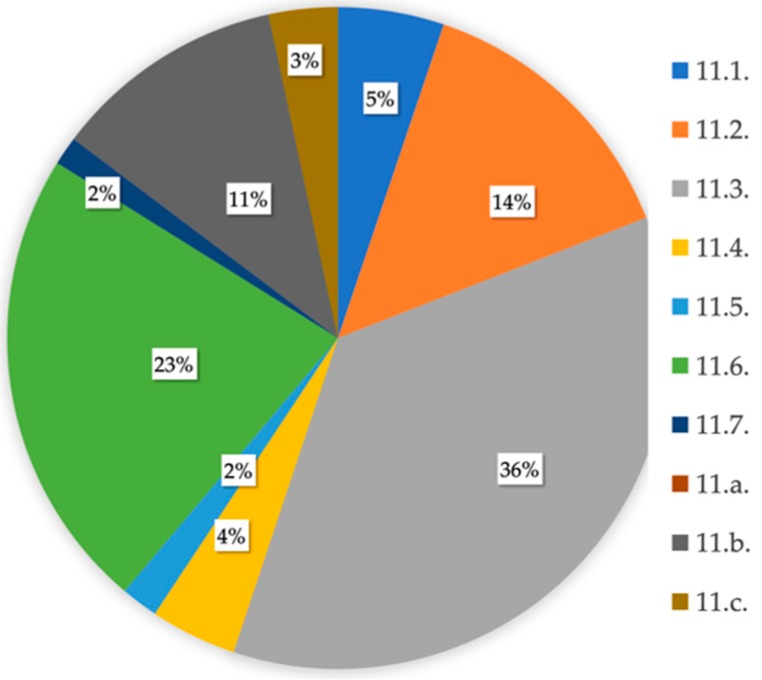
Proportion of actions associated with the 10 targets of SDG # 11 (Source: author).

The advocacy for an inclusive and sustainable urbanization as well as the participatory, integrated and sustainable human settlement planning and management are the subject of 11.3., which obtained the highest number of actions among all districts. Second, the reduction in the environmental impact of cities is examined by 11.6. Strengthening of social, economic, and environmental links between urban, peri-urban and rural areas (11.a.), universal access to safe green and public areas (11.7.), and the decrease in disaster fatalities (11.5.) displayed the lowest amount of effort adopted by the boroughs (Figure 8). The trend outlined by the link between the actions and targets of SDG # 11 was similar to that of total actions (Figure 4). Hence, the district with the largest amount of efforts included in its action plan (Tetuan) showed the strongest connection with the targets referred. In contrast, Ciudad Lineal and Barajas exhibited the weakest linkage with the SDG # 11 targets.

**Figure 9:**
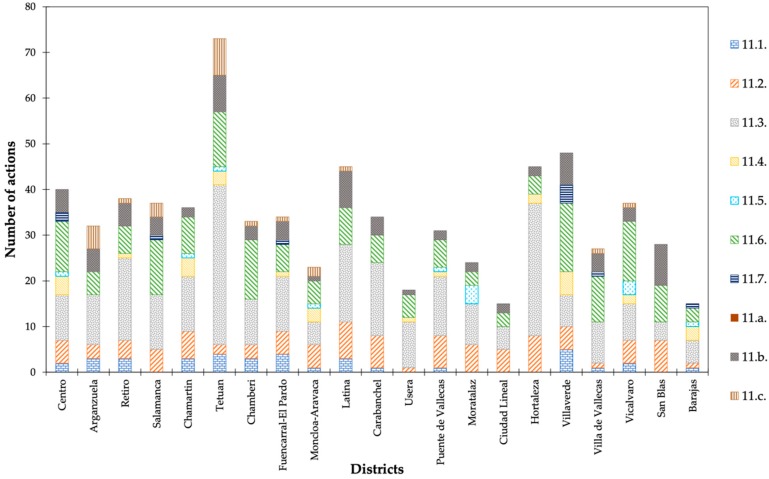
Assignment of actions to the boroughs and targets of SDG # 11 (Source: author).

Villaverde assigned a larger number of efforts to 11.1. (“Access for all to decent housing and basic services”), 11.4. (“Protect and safeguard world’s cultural and natural heritage”), 11.6. (“Reduction of adverse environmental impact of cities”), and 11.7. (“Universal access to safe green and public areas”). Targets 11.3. (“Participatory and integrated planning and management”) and 11.c. (“Financial and technical assistance to build sustainable and resilient buildings”) continued to show the same pattern in Tetuan. San Blas, Hortaleza and Latina, and Moratalaz reflected the closest relationship with 11.b. (“Promotion of resource efficiency and mitigation to climate change”), 11.2. (“Provide affordable, accessible and sustainable transport systems for all”), and 11.5. (“Reduction of Disaster fatalities”), respectively (Figure 9). 

## 4. Conclusions

The research analyzed the Local Agenda 21 of Madrid through a comparison with the Sustainable Development Goals, the global effort adopted by most countries in the framework of the 2030 Agenda to be reached by the end of 2030. The 3712 initiatives deemed in the action plans of the 21 districts of Madrid were correlated with the sustainability dimensions (social, economic, environmental and institutional), the 17 SDGs, and the 10 targets of SDG # 11 (“Sustainable cities and communities”). The main conclusions drawn from this study are listed below:

- The delay in the application of global actions by local and national governments leads to an undermining of their effectiveness, as they become outdated due to the ongoing deployment of new international measures. LA21 was carried out in Madrid between 2003 and 2013 when Agenda 21 was adopted in 1992 and further global initiatives have also been implemented.
- Effects of LA21 action plans on the sustainable urban development of Madrid remain unknown due to the absence of an integrated assessment framework comprising of metrics, an initial baseline and the goals to be reached.
- Urban, peri-urban, and rural areas bordering Madrid received no benefit from the LA21 process due to the lack of social, economic, and environmental linkages among them.
- Despite the organization of the city of Madrid by districts that should facilitate the access of citizens to municipal government, the public participation was limited to their minor role in the development of the LA21 process with scarce relevance in the shaping and approval of action plans.

Due to the parallelism in terms of methodology used between LA21 and the new Spanish Urban Agenda, some recommendations extracted from the results can be summarized as follows, to be applied during the implementation of the new Agenda:

- Sustainability should be embedded in local policies and funded through budgetary allocations for the long term to prevent its dependence on specific actions powered by national authorities such as Local Agenda 21 or the Spanish Urban Agenda.
- Although the Spanish Urban Agenda was defined as a tool to reflect each particular local context, a national framework should be created to provide common guidelines to all Spanish municipalities in order to share results and lessons learned.
- The development of strong liaisons with adjacent areas to municipalities where the Spanish Urban Agenda is applied could trigger high leverage effects therein.
- As a consequence of the changing nature of social, economic, and environmental aspects in the urban realm, the Spanish Urban Agenda should be conceived as a flexible framework to be periodically reviewed.
- An initial baseline, suitable metrics, and measurable goals should be determined to enable the evaluation and monitoring of the progress.
- Education and training on sustainable urban development for the key stakeholders involved in the Spanish Urban Agenda (citizens, local officials, and politicians) could stimulate civic participation and ensure the effectiveness in the process.
- The role of citizens in the new initiative should be strengthened by incorporating them into the decision-making processes.

Two major constraints limited this study. The paucity of information related to the advance of actions evaluated and the absence of metrics hampered any accurate assessment of the contribution of the LA21 process implemented in Madrid in the sustainable development of the city. As an extension of this work, a common framework that is deemed as a guideline of the new Spanish Urban Agenda is planned to be developed for its application in the main municipalities of the region of Cantabria. 

## Figures and Tables

**Table 1:** Classification of actions regarding the sustainability dimensions (Source: author).

District	Social	Economic	Environmental	Institutional	Total
Centro	41	13	51	97	202
Arganzuela	64	11	38	41	154
Retiro	53	7	45	51	156
Salamanca	55	11	39	81	186
Chamartin	73	7	35	57	172
Tetuan	148	13	51	146	358
Chamberi	55	8	34	49	146
Fuencarral-El Pardo	72	6	25	41	144
Moncloa-Aravaca	64	2	29	26	121
Latina	78	18	46	57	199
Carabanchel	68	18	30	60	176
Usera	58	13	23	21	115
Puente de Vallecas	64	11	21	83	179
Moratalaz	47	12	15	34	108
Ciudad Lineal	44	2	18	5	69
Hortaleza	110	13	29	122	274
Villaverde	103	22	65	120	310
Villa de Vallecas	77	14	46	53	190
Vicalvaro	97	15	53	77	242
San Blas	59	7	39	24	129
Barajas	48	1	15	18	82
Total	1478	224	747	1263	3712

**Table 2:** Relationship between the SDGs and Aalborg Commitments [3,10].

SDG #	Sustainable Development Goal (SDG)	Corresponding AC
1	No poverty	
2	Zero hunger	
3	Good health and well-being	7
4	Quality education	
5	Gender equality	
6	Clean water and sanitation	
7	Affordable and clean energy	
8	Decent work and economic growth	8
9	Industry, innovation and infrastructure	
10	Reduced inequalities	9
11	Sustainable cities and communities	5, 6
12	Responsible consumption and production	3, 4
13	Climate action	
14	Life below water	
15	Life on land	
16	Peace, justice and strong institutions	2, 10
17	Partnerships for the goals	1

**Table 3:** Distribution of actions among the SDGs (Source: author).

District/SDG #	# 1	# 2	# 3	# 4	# 5	# 6	# 7	# 8	# 9	# 10	# 11	# 12	# 13	# 14	# 15	# 16	# 17	Total District
Centro	1	0	22	9	6	6	6	24	11	8	53	7	1	0	0	16	32	202
Arganzuela	0	0	27	17	2	2	8	12	7	7	11	12	1	0	3	12	33	154
Retiro	0	0	13	11	1	5	5	7	1	5	14	10	1	0	8	23	52	156
Salamanca	0	0	27	16	4	4	3	11	1	9	29	14	6	0	3	15	44	186
Chamartin	0	0	23	11	4	3	3	9	7	20	21	10	3	0	3	12	43	172
Tetuan	0	0	43	41	22	6	6	13	6	41	39	14	6	0	7	41	73	358
Chamberi	0	0	17	14	0	4	5	10	2	3	28	10	3	0	9	7	34	146
Fuencarral-El Pardo	0	0	21	20	0	2	6	7	6	11	21	5	1	0	5	11	28	144
Moncloa-Aravaca	0	0	14	17	3	3	4	5	3	18	14	5	1	2	5	10	17	121
Latina	0	0	13	32	3	5	4	17	5	12	28	10	5	0	4	19	42	199
Carabanchel	0	0	22	22	6	3	5	19	4	13	23	6	4	0	5	20	24	176
Usera	0	0	13	16	3	3	1	17	9	12	18	7	1	0	2	3	0	115
Puente de Vallecas	0	0	24	23	2	2	2	10	3	16	28	7	0	0	4	12	48	179
Moratalaz	0	0	18	12	1	4	0	11	6	6	27	2	0	0	4	15	2	108
Ciudad Lineal	0	0	11	9	1	4	0	2	10	2	11	3	0	0	5	10	1	69
Hortaleza	0	0	40	29	0	4	1	6	8	23	48	8	0	0	12	25	70	274
Villaverde	0	0	36	36	8	12	6	17	8	18	41	16	2	2	17	20	71	310
Villa de Vallecas	0	0	36	12	3	3	4	12	6	20	30	17	3	0	3	13	28	190
Vicalvaro	0	0	23	32	4	4	5	15	11	21	36	18	5	0	11	16	41	242
San Blas	0	0	22	7	4	16	4	7	3	14	16	7	0	0	7	5	17	129
Barajas	0	0	22	12	0	0	0	3	6	5	13	3	1	0	6	8	3	82
Total	1	0	487	398	77	95	78	234	123	282	549	191	44	4	123	313	703	3712

**Table 4:** Description of the targets included in Sustainable Development Goal # 11 [3].

Target 11.#.	Target of Sustainable Development Goal # 11
11.1.	“Ensure access for all to adequate, safe and affordable housing and basic services and upgrade slums”
11.2.	“Provide access to safe, affordable, accessible and sustainable transport systems for all, improving road safety, notably by expanding public transport, with special attention to the needs of those in vulnerable situations, women, children, persons with disabilities and older persons”
11.3.	“Enhance inclusive and sustainable urbanization and capacity for participatory, integrated and sustainable human settlement planning and management in all countries”
11.4.	“Strengthen efforts to protect and safeguard the world’s cultural and natural heritage”
11.5.	“Significantly reduce the number of deaths and the number of people affected and substantially decrease the direct economic losses relative to global gross domestic product caused by disasters, including water-related disasters, with a focus on protecting the poor and people in vulnerable situations”
11.6.	“Reduce the adverse per capita environmental impact of cities, including by paying special attention to air quality and municipal and other waste management”
11.7.	“Provide universal access to safe, inclusive and accessible, green and public spaces, in particular for women and children, older persons and persons with disabilities”
11.a.	“Support positive economic, social and environmental links between urban, peri-urban and rural areas by strengthening national and regional development planning”
11.b.	“Substantially increase the number of cities and human settlements adopting and implementing integrated policies and plans towards inclusion, resource efficiency, mitigation and adaptation to climate change, resilience to disasters, and develop and implement, in line with the Sendai Framework for Disaster Risk Reduction 2015-2030, holistic disaster risk management at all levels”
11.c.	“Support least developed countries, including through financial and technical assistance, in building sustainable and resilient buildings utilizing local materials”

**Table 5:** Sharing of actions between targets of SDG # 11 (Source: author).

District	11.1.	11.2.	11.3.	11.4.	11.5.	11.6.	11.7.	11.a.	11.b.	11.c.	Total	Total District
Centro	2	5	10	4	1	11	2	0	5	0	40	202
Arganzuela	3	3	11	0	0	5	0	0	5	5	32	154
Retiro	3	4	18	1	0	6	0	0	5	1	38	156
Salamanca	0	5	12	0	0	12	1	0	4	3	37	186
Chamartin	3	6	12	4	1	8	0	0	2	0	36	172
Tetuan	4	2	35	3	1	12	0	0	8	8	73	358
Chamberi	3	3	10	0	0	13	0	0	3	1	33	146
Fuencarral-El Pardo	4	5	12	1	0	6	1	0	4	1	34	144
Moncloa-Aravaca	1	5	5	3	1	5	0	0	1	2	23	121
Latina	3	8	17	0	0	8	0	0	8	1	45	199
Carabanchel	1	7	16	0	0	6	0	0	4	0	34	176
Usera	0	1	10	1	0	5	0	0	1	0	18	115
Puente de Vallecas	1	7	13	1	1	6	0	0	2	0	31	179
Moratalaz	0	6	9	0	4	3	0	0	2	0	24	108
Ciudad Lineal	0	5	5	0	0	3	0	0	2	0	15	69
Hortaleza	0	8	29	2	0	4	0	0	2	0	45	274
Villaverde	5	5	7	5	0	15	4	0	7	0	48	310
Villa de Vallecas	1	1	9	0	0	10	1	0	4	1	27	190
Vicalvaro	2	5	8	2	3	13	0	0	3	1	37	242
San Blas	0	7	4	0	0	8	0	0	9	0	28	129
Barajas	1	1	5	3	1	3	1	0	0	0	15	82
Total	37	99	257	30	13	162	10	0	81	24	713	3712
